# Supplementary material for: WordCommentsAnalyzer: A windows software tool for qualitative research
Source: F1000Res. 2018 Sep 4;7:536. Originally published 2018 May 3. [Version 2] doi: 10.12688/f1000research.14819.2 (PMC6137411; doi:10.12688/f1000research.14819.2)
Supplement: Supplementary file 3 [file f1000research-7-17614-s0002.tgz › b2a79496-4ada-47c4-9682-a99ce867836d.zip!/Eight Iranian Health Journals/Journal of Health System Research _7.docx]

© Journal of Health System Research . This work is licensed under CC BY-NC 4.0 (https://creativecommons.org/licenses/by-nc/4.0/).

Volume & Issue: دوره 12، شماره 3، 1395

Title: بررسی کارایی فرایند انعقاد و لخته‌سازی شیمیایی به وسیله منعقد کننده‌های مختلف (آلوم، کلرورفریک، پلی‌ آلومینیوم کلراید و آهک) در حذف رنگ Navy Blue CE-RN نساجی از محلول‌های آبی

http://hsr.mui.ac.ir/index.php/jhsr/article/view/1928

مقدمه: مطالعه حاضر به بررسی کارایی منعقد کننده‌های آلوم (Al2(SO4)3.18 H2O)، کلرورفریک (FeCl3.6H2O)، پلی آلومینیوم کلراید (Polyaluminium Chloride یا PAC) و آهک (CaO) جهت حذف رنگ Navy Blue CE-RN نساجی از محلول‌های آبی پرداخت. روش‌ها: خصوصیات رنگ شامل طول موج غالب، ته رنگ، درجه درخشندگی و درصد خلوص رنگ Navy Blue CE-RN با روش Multi-wavelength spectrophotometric تعیین گردید. دوز و pH بهینه و SVI (Sludge volume index) منعقد کننده‌ها از طریق آزمایش Jar بررسی شد. در نهایت، کارایی منعقد کننده‌ها در شرایط بهینه با یکدیگر مقایسه گردید. انتخاب منعقد کننده بهتر با توجه به کارایی و مسایل اقتصادی صورت گرفت. یافته‌ها: طول موج غالب، درجه درخشندگی و درصد خلوص رنگ طبق روش ذکر شده به ترتیب nm 473، 19 درصد و 53 درصد به دست آمد. PAC بهترین کارایی را در بین منعقد کننده‌ها با دوز 1/0 گرم بر لیتر و pH بهینه 6 در حذف COD (Chemical oxygen demand) و رنگ به ترتیب با راندمان 84 و 93 درصد داشت و SVI آن برابر با 1324 لیتر بر گرم بود. نتیجه‌گیری: نتایج حاصل از پژوهش نشان داد که PAC، یک منعقد کننده با کارایی بالا و صرفه اقتصادی در حذف رنگ Navy Blue CE-RN است

Title: بررسی نگرش و رفتار مردم شهر اصفهان در خصوص اهدای اعضا

http://hsr.mui.ac.ir/index.php/jhsr/article/view/2158

مقدمه: بهبود نگرش و عملكرد جامعه با هدف تغيير باورهاي نادرست مي‌تواند بر روی افزايش اهداي اعضا پس از مرگ مغزي مؤثر باشد. مطالعه حاضر جهت تعیین نگرش و عملكرد مردم شهر اصفهان در خصوص اهداي اعضا صورت گرفت. روش‌ها: این مطالعه از نوع توصیفی بود. 975 نفر از افراد بالای 15 سال، جامعه مورد مطالعه را تشکیل دادند که با روش نمونه‌گیری تصادفی طبقه‌ای از 10 منطقه شهر اصفهان انتخاب شدند. ابزار جمع‌آوری اطلاعات شامل پرسش‌نامه خود ساخته دارای 2 بخش اطلاعات زمینه‌ای و سؤال‌های نگرش و رفتار بود. داده‌ها با استفاده از آزمون‌هاي Independent t و آناليز واريانس در نرم‌افزار SPSS نسخه 17 مورد تجزيه و تحليل قرار گرفت و سطح معنی‌داری کمتر از 05/0 در نظر گرفته شد. يافته‌ها: میانگین نمره نگرش و رفتار شرکت کنندگان به ترتیب برابر 6 ± 8/36 و 2/0 ± 47/0 (از 1 نمره) به دست آمد كه در حد مطلوب بود. جنسیت نقش مؤثری بر سطح نگرش افراد داشت. سطح تحصيلات نیز نقش مؤثری بر سطح نگرش و عملكرد فرد در خصوص اهداي عضو نشان داد (05/0 P <). در نهايت، نگرش افرادي بالاتر بود كه رفتار مطلوب داشتند. اين تفاوت از نظر آماري معنی‌دار به دست آمد (05/0 P <). نتيجه‌گيري: مطالعه حاضر نشان داد كه نگرش و عملكرد مردم شهر اصفهان در خصوص اهداي عضو در حد متوسط است و نگرش در افرادي كه رفتار مطلوب داشتند، بالاتر بود. از این‌رو، مي‌توان با آموزش‌هاي عمومي در سطح جامعه و ايجاد نگرش مثبت، ميزان اهدا و به تبع آن ميزان پيوند را افزايش داد.

Title: بررسی وضعیت بهداشت محیط مدارس ابتدایی دولتی دخترانه خمینی‌شهر اصفهان در سال 1392

http://hsr.mui.ac.ir/index.php/jhsr/article/view/2260

مقدمه: هدف از مطالعه حاضر، بررسی وضعیت بهداشت محیط مدارس ابتدایی دولتی دخترانه خمینی‌شهر اصفهان در سال 1392 بود. روش‌ها: مطالعه حاضر، از نوع توصیفی- مقطعی بوده که از طریق تکمیل چک‌لیست و سنجش میدانی در 12 مدرسه دولتی دخترانه خمینی‌شهر اصفهان انجام گردید. درجه حرارت و اندازه کلاس‌های درس با استفاده از یک دماسنج و یک نوار اندازه‌گیری شد. یک لوکس متر دیجیتال مدل TES1332A برای تعیین روشنایی و انعکاس کلاس درس استفاده شد. آزمون مقایسه میانگین با عدد ثابت (One sample t-test) برای تجزیه و تحلیل نتایج به دست آمده مورد استفاده قرار گرفت. یافته‌ها: در این مطالعه، 25 درصد از کلاس‌ها از روشنایی مناسب برخوردار بود. در مورد کتابخانه این عدد به 3/33 درصد رسید. هیچ یک از توالت و دستشویی‌ها دارای روشنایی مناسب نبود. حدود 7/66 درصد از مدارس از نظر تعداد توالت با استاندارد مربوط مطابقت داشت. تعداد آبخوری‌های کل مدارس مطابق با استاندارد بود. حدود 7/91 درصد از مدارس فضای مناسب برای هر دانش‌آموز داشت، ولی سطح زمین بازی به ازای هر نفر در 7/91 درصد از مدارس رعایت نشده بود. وضعیت دیوار کلاس‌ها و راهروها از نظر ارتفاع نصب سنگ در هیچ یک از مدارس مطابق استاندارد نبود. نتیجه‌گیری: با توجه به نتایج حاصل، در بعضی از مدارس وضعیت نیمکت‌ها، شدت روشنایی کلاس‌ها، راهروها، دستشویی و توالت، کتابخانه، سطح زمین بازی، میزان انعکاس نور در کلاس‌ها، فاصله آبخوری تا دستشویی، وضعیت دیوار کلاس‌ها و وضعیت تفکیک زباله در حد مطلوب نبود که هماهنگی بیشتر بین واحد بهداشت مدارس معاونت بهداشتی استان، سازمان آموزش و پرورش و توجه بیشتر آن‌ها در جهت رفع این مشکلات را نیازمند است.

Title: تأثیر آموزش بر آگاهی، موانع درک شده، وضعیت و تداوم مراجعه بیماران به بخش بازتوانی قلب بیمارستان شهید چمران اصفهان

http://hsr.mui.ac.ir/index.php/jhsr/article/view/2295

مقدمه: بیماری‌های قلبی از مهم‌ترین دلایل مرگ می‌باشد و موجب کاهش کیفیت زندگی می‌گردد. خدمات بازتوانی باعث ارتقای سطح سلامت این بیماران می‌شود، اما وضعیت استفاده از این خدمات مطلوب نیست. مطالعه حاضر با هدف بررسی تأثیر آموزش بر آگاهی، موانع درک شده و وضعیت مراجعه بیماران به بخش بازتوانی قلب بیمارستان شهید چمران اصفهان اجرا شد. روش‌ها: این مطالعه نیمه تجربی بر روی 69 نفر بيمار بستری (گروه مداخله 35 نفر و گروه شاهد 34 نفر) بيمارستان شهید چمران اصفهان در سال 1392 صورت گرفت. بیماران واجد شرایط به طور تصادفی به یکی از دو گروه اختصاص یافتند. داده‌ها با استفاده از پرسش‌نامه محقق ساخته و مورد تأیید کارشناسان جمع‌آوری شد. آموزش گروه مداخله به صورت چهره به چهره، تلفنی، پمفلت، فیلم و اسلاید بود. وضعیت مراجعه و تعداد دفعات حضور بیماران در برنامه بازتوانی از طریق پیگیری تلفنی و بررسی لیست مراجعه کنندگان دو ماه بعد از اولین مراجعه بیماران به واحد بازتوانی بررسی گردید. داده‌ها با استفاده از آزمون‌های مناسب در نرم‌افزار SPSS با سطح معنی‌داری 05/0 مورد تجزیه و تحلیل قرار گرفت. یافته‌ها: میانگین سن بیماران گروه مداخله و شاهد به ترتیب 1/2 ± 57/58 و 8/1 ± 94/55 سال به دست آمد. افزایش و کاهش معنی‌داری پس از مداخله به ترتیب در آگاهی (01/0 > P) و موانع درک شده (01/0 > P) بیماران نسبت به گروه شاهد ایجاد شد. دفعات مراجعه بیماران گروه مداخله نسبت به گروه شاهد برای بازتوانی بیشتر بود (03/0 > P). بیمارانی که به واحد بازتوانی مراجعه می‌کردند، میانگین نمره آگاهی بیشتر (01/0 > P) و میانگین نمره موانع درک شده کمتری (03/0 = P) نسبت به سایرین داشتند. نتیجه‌گیری: مداخله آموزشی با هدف افزایش آگاهی نسبت به اثرات مفید برنامه‌های بازتوانی و کاهش موانع درک شده بیماران جهت شرکت در این برنامه‌ها، توانست تداوم مراجعه بیماران را به بخش بازتوانی افزایش دهد.

Title: مقایسه فعالیت فتوکاتالیستی نانو ذرات اکسید روی تولیدی و تجاری جهت حذف رنگ در محیط آبی

http://hsr.mui.ac.ir/index.php/jhsr/article/view/2413

مقدمه: هدف از پژوهش حاضر، تولید نانو ذرات اکسید روی و مقایسه فعالیت فتوکاتالیستی آن با نوع تجاری جهت اکسيداسيون فتوکاتاليستي رنگزای متیلن بلو در محیط آبی بود. روش‌ها: ابتدا نانو ذرات اکسید روی با استفاده از تیمار پراکسید هیدروژن تهیه شد و با استفاده از تثبیت حرارتی بر روی سطح شیشه تثبیت گردید. شیشه‌های آماده شده در داخل راکتور قرار گرفت. در نهایت، تجزیه فتوکاتالیستی متیلن بلو در این راکتور با متغیرهای مختلف بررسی شد. یافته‌ها: فرایند فتوکاتالیستی با کاتالیست تولید شده (35/96 درصد) در قیاس با کاتالیست تجاری (87/82 درصد) جهت حذف رنگزای متیلن بلو با غلظت 5 میلی‌گرم بر لیتر در مدت زمان 120 دقیقه راندمان بیشتری داشت. در ادامه، با استفاده از کاتالیست تولید شده، در فواصل 2، 5/3 و 5 سانتیمتر بین لامپ‌های فرابنفش و صفحات شیشه‌ای حاوی کاتالیست، میزان حذف رنگ به ترتیب 35/96، 34/63 و 31/45 درصد به دست آمد. نتیجه‌گیری: کاتالیست نانو ذرات اکسید روی تولید شده می‌تواند به عنوان یک کاتالیست مؤثر جهت فرایندهای فتوکاتالیستی به کار گرفته شود.

Title: ارزیابی خطر ابتلا به اختلالات اسکلتی- عضلانی در بخش‌های بیمارستانی با استفاده از شاخص MAPO

http://hsr.mui.ac.ir/index.php/jhsr/article/view/2278

مقدمه: اختلالات اسکلتی- عضلانی از مشکلات بهداشتی مهم در پرستاران است. از جمله دلایل مهم در کارکنان پرستاری وظایف جابجایی بیمار می‌باشد. هدف از انجام مطالعه حاضر، بررسی خطر ابتلا به اختلالات اسکلتی- عضلانی در بخش‌های بیمارستانی با استفاده از شاخص MAPO (Movement and assistance of hospital patient)  بود. روش‌ها: اين مطالعه به صورت توصيفي و مقطعی در بین 200 نفر از پرسنل پرستار شاغل در 30 بخش، 5 بیمارستان مورد بررسي قرار گرفت. داده‌ها با استفاده از پرسش‌نامه‌هاي ويژگي‌هاي دموگرافيك، Nordic و چك‌لیست شاخص MAPO جمع‌آوری شد. تجزيه و تحليل داده‌ها با استفاده از نرم‌افزار SPSS و Excel انجام شد. یافته‌ها: ميانگين ساعات كار در روز برای هر دو گروه مرد و زن 46/0 ± 31/7 ساعت بود. بر اساس یافته‌ها، بین جنس، سن، سابقه کاری، شغل، BMI (Body mass index) و ورزش کردن با اختلالات مربوط به اندام‌ها از لحاظ آماری رابطه معنی‌داری وجود داشت (05/0 > P). در شاخص MAPO، 1/21 درصد از افراد مورد بررسی در ناحیه 1 یا ریسک خطر قابل اغماض یا چشم‌پوشی، 6/52 درصد در ناحیه 2 یا برنامه‌های مداخله‌ای میان مدت و بلند مدت و در نهایت، 3/26 درصد در ناحیه 3 یا مداخله‌ای فوری و بهبود شرایط محیطی قرار داشتند. نتیجه‌گیری: میزان شیوع اختلالات اسکلتی- عضلانی در ميان پرسنل پرستاري بالا است. جهت کاهش اختلالات مذکور، پيشنهاد مي‌گردد که تجهیزات مدرن لازم برای جابجایی بیماران خریداری و نحوه صحیح استفاده از این تجهیزات به پرسنل آموزش داده شود و تعداد نیروی کافی در هر بخش استخدام گردد تا از وارد آمدن فشار ناشی از کمبود نیروی کاری بر افراد کاسته شود.

Title: مقایسه کیفیت زندگی بیماران مبتلا به سرطان در استرالیا و ایران: یک مطالعه بین فرهنگی

http://hsr.mui.ac.ir/index.php/jhsr/article/view/2429

مقدمه: مقایسه کیفیت زندگی بیماران مبتلا به سرطان بین دو فرهنگ می‌تواند نکات ارزشمندی را در ارتباط با نقاط قوت و ضعف احتمالی ارایه مراقبت به این بیماران و راهکارهای ارتقای کیفیت زندگی آنان فراهم کند. با توجه به عدم بازیافت مطالعات بین فرهنگی در این زمینه، تحقیقی با هدف مقایسه کیفیت زندگی بین بیماران مبتلا به سرطان استرالیایی و ایرانی در ابعاد چهارگانه (جسمی، روانی، اجتماعی و محیطی) طراحی شد. روش‌ها: این مطالعه بین فرهنگی از نوع توصیفی بود که اطلاعات مرتبط با کیفیت زندگی 166 بیمار مبتلا به سرطان سه بیمارستان مرجع استرالیا با اطلاعات کیفیت زندگی 198 بیمار مبتلا به سرطان یک بیمارستان مرجع ایران مقایسه گردید. هر دو گروه از بیماران پرسش‌نامه کیفیت زندگی خلاصه سازمان بهداشت جهانی (World Health Organization Quality of Life-Bref یا WHOQOL-BREF) را تکمیل کردند. یافته‌ها: شایع‌ترین سرطان‌ در مراکز منتخب استرالیا به ترتیب شامل سرطان سینه، لنفوم غیر هوچکینی، کولورکتال، ریه و لوسمی و در ایران شامل سرطان لوسمی، کولورکتال، لنفوم غیر هوچکینی و سینه بود. میانگین نمره کیفیت زندگی بیماران استرالیایی به طور معنی‌داری بالاتر از میانگین نمره کیفیت زندگی بیماران ایرانی در تمامی حیطه‌ها به دست آمد. نتیجه‌گیری: پایین بودن کیفیت زندگی بیمارن مبتلا به سرطان در ایران نسبت به استرالیا می‌تواند سیاست‌گذاران و مراقبان بهداشتی را تشویق نماید تا برای فراهم کردن زیرساخت‌ها و منابع جهت ارتقای کیفیت زندگی بیماران تلاش کنند.

Title: بررسی آلودگی منابع آب زیرزمینی دشت قلعه شاهین به عناصر سرب و کادمیوم با استفاده از شاخص آلودگی فلزات سنگین (93-1392)

http://hsr.mui.ac.ir/index.php/jhsr/article/view/2529

مقدمه: امروزه به دلیل تخلیه انواع آلاینده‌ها به منابع آب‌های زیرزمینی، این منابع با افت کیفیت مواجه شده‌اند. از این‌رو، مطالعه حاضر جهت ارزیابی کیفی آلودگی منابع آب زیرزمینی دشت قلعه شاهین به سرب و کادمیوم، با استفاده از شاخص آلودگی فلزات سنگین (HPI یا Heavy metal pollution index) در طول زمستان 1392 و تابستان 1393 انجام گردید. روش‌ها: در این مطالعه توصیفی، نمونه‌ها از 20 ایستگاه منتخب بر اساس دستورالعمل 3010-B، جمع‌آوری گردید. پس از آماده‌سازی نمونه‌ها در آزمایشگاه، غلظت فلزات با استفاده از دستگاه ICP-OES تعیین شد. ارزیابی کیفی منابع آب زیرزمینی و تجزیه و تحلیل آماری با استفاده از شاخص HPI و نرم‌افزار آماری SPSS انجام شده است. یافته‌ها: میانگین غلظت سرب و کادمیوم بر حسب ppb به ‌ترتیب در نمونه‌های فصل زمستان برابر با 47/1 ± 25/4 و 62/0 ± 48/0 و در نمونه‌های فصل تابستان برابر با 22/2 ± 46/6 و 36/2 ± 21/1 بود. همچنین، میانگین مقادیر شاخص HPI مربوط به نمونه‌های فصول زمستان و تابستان به ترتیب برابر با 73/4 و 74/11 بود که بسیار کم‌تر از مقادیر بحرانی آلودگی آب آشامیدنی بود. نتیجه‌گیری: گرچه در حال حاضر منابع آب زیرزمینی دشت قلعه شاهین به عناصر سرب و کادمیوم آلوده نیست، اما با توجه به توان کشت دو بار در سال در منطقه مورد مطالعه، نظارت بر منابع آب زیرزمینی این منطقه به صورت دوره‌ای از نظر غلظت فلزات سنگین توصیه می‌شود.

Title: مکان‌یابی محل بهینه دفع پسماند ساختمانی با استفاده از روش ترکیب خطی وزنی، مطالعه موردی شهر اصفهان (1393)

http://hsr.mui.ac.ir/index.php/jhsr/article/view/2535

مقدمه: از پیامدهای توسعه روزافزون فضاهای شهری، تولید انبوه پسماند شهری از جمله پسماندهای ناشی از فعالیت‌های ساختمانی است. از این‌رو، برنامه‌ریزی منسجم مبتنی بر اصول مدیریتی و مهندسی برای ساماندهی این گونه از پسماندها ضروری به نظر می‌رسد. از آن‌جایی که هیچ گونه مطالعات قبلی در رابطه با مکان‌یابی اصولی و بهینه محل دفع این پسماندها در شهر اصفهان با توجه به معیارهای موجود صورت نگرفته است، در مطالعه حاضر، میزان مطلوبیت اراضی شهر اصفهان جهت دفع پسماندهای ساختمانی این شهر، با استفاده از روش ترکیب خطی وزنی و به کمک سیستم اطلاعات جغرافیایی بررسی شد. روش‌ها: مطالعه حاضر از نوع کاربردی توسعه‌ای بود. به منظور شناسایی بهترین مکان برای دفع پسماند ساختمانی شهر اصفهان، 19 لایه اطلاعاتی در محدوده شهر اصفهان در دو شاخه اصلی پارامترهای اکولوژیکی و اقتصادی- اجتماعی دسته‌بندی شد. سپس، به منظور وزن‌دهی هر لایه، از نظرات کارشناسی در قالب ماتریس مقایسات زوجی (روش تحلیل سلسله مراتبی) استفاده شد. همچنین، استانداردسازی لایه‌های معیار با استفاده از منطق فازی و تعریف توابع عضویت فازی مناسب برای هر معیار انجام گرفت و در نهایت، نقشه مطلوبیت منطقه برای دفع پسماند به کمک توابع همپوشانی نقشه‌های استاندار شده وزن‌دار و نیز 16 لایه محدودیت به ‌دست آمد. یافته‌ها: با توجه به نتایج حاصل از طبقه‌بندی نقشه نهایی مطلوبیت محل دفع پسماند، بیش از 95 درصد از سطح منطقه مورد مطالعه از وضعیت کامل نامطلوب برای دفع پسماند ساختمانی برخوردار بوده و محدوده‌های با مطلوبیت بسیار زیاد کمتر از 2 درصد از مساحت شهر را به خود اختصاص داد که در نهایت، از بین این مناطق، 4 محدوده به عنوان گزینه‌های پیشنهادی برای محل دفع پسماند ساختمانی شهر اصفهان شناسایی و معرفی شد. نتیجه‌گیری: توانایی و سودمندی سامانه اطلاعات جغرافیایی در پیاده‌سازی روش‌های تصمیم‌گیری چند معیاره برای مکان‌یابی محل دفع پسماندهای شهری و ترکیب معیارهای مختلف کمی و کیفی نشان داده شد. معرفی 78/1 درصد از مساحت کل منطقه به عنوان منطقه بسیار مناسب برای محل دفع نشان ‌دهنده حساسیت و دقت بالای مدل ((WLC weight line composition بوده و نیز محدودیت منطقه را برای دفع پسماند متذکر می‌شود.

Title: بررسی تأثیر سطوح مختلف ویتامین D3، عصاره روغنی زیره سبز و زمان تخمیر با استفاده از روش سطح پاسخ بر بهینه‌سازی تولید ماست پروبیوتیک برای بیماران دیابتی

http://hsr.mui.ac.ir/index.php/jhsr/article/view/2750

مقدمه: در سال‌های اخیر، فواید دریافت پروبیوتیک‌ها، ویتامین D3 و اسانس روغنی زیره سبز، هر کدام به تنهایی در مدیریت بیماری دیابت مورد توجه قرار گرفته است. در مطالعه حاضر، تولید ماست حاوی غلظت‌های مختلفی از ویتامین D3 و اسانس روغنی زیره سبز با به کار بردن زمان‌های مختلف تخمیر برای دستیابی به محصول پروبیوتیک با بالاترین شمارش سلولی باکتری پروبیوتیک که به شکل کشت الحاقی به آن افزوده شده بود، مورد بررسی قرار گرفت و تولید فراورده پروبیوتیک با ارزش سلامتی افزوده برای کمک به افراد دیابتی هدف نهایی مطالعه تعیین شد. روش‌ها: روش سطح پاسخ با مدل مرکب مرکزی برای بررسی تأثیر سه فاکتور متفاوت شامل اسانس روغنی زیره، ویتامین D3 و زمان تخمیر بر جمعیت سویه پروبیوتیک باکتری لاکتوباسیلوس پلانتاروم A7 مورد استفاده قرار گرفت. غلظت اسانس زیره در چهار سطح 01/0، 02/0، 03/0 و 05/0 درصد و غلظت ویتامین D در پنج سطح 20، 40، 400، 1000 و 2000 IU و طول زمان تخمیر در پنج سطح 3، 6، 9، 12 و 24 ساعت متغیرهای آزمایش تعریف شد. بر اساس مدل مورد استفاده، 15 طرح آزمایشی در 20 تکرار تعیین شد و نتایج در نرم‌افزار SAS (Statistical analysis system) مورد تجزیه و تحلیل قرار گرفت. تأثیر هر یک از متغیرهای مورد بررسی بر نتیجه نهایی در سطح 5 درصد احتمال معنی‌دار در نظر گرفته شد (05/0 > P). یافته‌ها: اثر توأم غلظت اسانس زیره و زمان بر نرخ رشد باکتری لاکتوباسیلوس پلانتاروم A7 بیشترین اثر را داشت و به ترتیب بعد از آن اثر توأم زیره و ویتامین D3، ویتامین D3 با توان دو و ویتامین D3 با توان یک بر نرخ رشد بیشینه سویه باکتری واقع شد. بعد از آن، اسانس زیره با توان یک و زمان با توان یک اثر کمی بر نرخ رشد این باکتری نشان داد که شرایط بهینه برای فرمولاسیون حد میانی هر یک از متغیرها تعیین گردید. نتیجه‌گیری: ماست پروبیوتیک غنی شده با ویتامین D3 و اسانس زیره می‌تواند حاوی جمعیت بالایی از باکتری زنده (107cfu/ml) از سویه پروبیوتیک الحاقی باشد. این محصول می‌تواند به عنوان محصول فراسودمند برای افرادی که از بیماری دیابت رنج می‌برند، مورد استفاده قرار گیرد.

Title: عوامل مؤثر بر تکرار اقدام به خودکشی در جوانان و بزرگسالان اصفهانی: مدل‌های رگرسیونی شمارشی مثبت آماسیده- یک

http://hsr.mui.ac.ir/index.php/jhsr/article/view/2832

مقدمه: فاکتورهاي مخاطره از موارد مهم در پيشگيري از اقدام به خودکشي شناخته شده‌اند. هدف از پژوهش حاضر، بررسي فاکتورهاي مؤثر بر تکرار اقدام به خودکشي در جوانان و بزرگسالان است که توسط تعميمي از مدل‌هاي رگرسيوني شمارشي مثبت انجام گرفت. روش‌ها: اطلاعات 701 بيمار (183 جوان و 518 بزرگسال)، با ويژگي حداقل يک بار اقدام به خودکشي ناموفق، به روش DSM-IV (Diagnostic and statistical manual of mental disorders, 4th edition) در شهر اصفهان جمع‌آوري شد. برای تحليل داده‌ها علاوه بر ارایه شاخص‌های آمار توصيفی، از برازش مدل‌هاي رگرسيوني شمارشي مثبت با خاصيت آماسيده- يک نيز استفاده گردید. یافته‌ها: مدل Poisson مثبت آماسيده- يک براي گروه سني جوانان مناسب بود. فاکتورهاي سن و وجود پيشينه‌ خانوادگي داراي اثر مثبت و فاکتور اختلال سازگار در مقايسه با افسردگي اثر منفي در تکرار اقدام به خودکشي بين جوانان داشت. نتايج حاصل از برازش مدل دوجمله‌اي- منفي با ويژگي شمارشي مثبت و آماسيده- يک براي گروه سني بزرگسالان نشان داد که زندگي در ناحيه روستايي، وجود پيشينه‌ خانوادگي و تصميم‌گيري بدون طراحي قبلي داراي اثر مثبت بر تکرار اقدام به خودکشي بود. همچنين، تکرار اقدام به خودکشي در بيماران با اختلال دوقطبي نسبت به افراد افسرده عدد بيشتری را نشان داد. نتیجه‌گیری: در گروه سني جوانان فاکتورهاي اختلال سازگار و سن معنی‌دار بود؛ در صورتي که براي بزرگسالان اختلال دوقطبي و منطقه‌ سکونت از فاکتورهاي تأثيرگذار است. همچنين، در هر دو گروه سني وجود پيشينه خانوادگي اثر مثبت بر تکرار اقدام به خودکشي دارد.

Title: بررسی شاخص‌های سلامت سالمندان استان اصفهان سال 92-1390

http://hsr.mui.ac.ir/index.php/jhsr/article/view/2134

مقدمه: گرچه پیر شدن جمعیت از نتایج مثبت توسعه به شمار می آید، ولی اگر برای مواجهه با آن آماده نباشیم، عوارض و پیامدهای منفی بسیاری در پی خواهد داشت. بر همین اساس، مطالعه حاضر با هدف تعیین شاخص های سلامت سالمندان استان اصفهان به منظور بهره برداری در طراحی برنامه ها انجام شد. روش‌ها: نوع مطالعه، توصیفی از نوع مقطعی و روش نمونه‌گیری طبقه‌بندی خوشه ای تصادفی با حجم نمونه 4321 بود. اطلاعات بر اساس پرسش‌نامه های 36 سؤالی (36SF یا Short form 36 health survey) استاندارد، سبک زندگی سالم در سالمندان ایرانی و وضعیت سلامت با روایی و پایایی مورد تأیید جمع‌آوری گردید. اطلاعات توسط نرم‌افزار SPSS و به کمک روش های آمار توصیفی و t مورد تجزیه و تحلیل قرار گرفت. یافته‌ها: سالمندان در مطالعه حاضر 5/78 درصد بیمار بوده که اختلال خواب 8/62 درصد، فشار خون بالا 1/44 درصد و فراموشکاری 6/43 درصد بالاترین درصد بیماری ها را به خود اختصاص داد. از نظر سبک و کیفیت زندگی به ترتیب با میانگین نمرات 5/143 و 58 وضعیت مردان بهتر از زنان بود. نتیجه‌گیری: با توجه به اطلاعات حاصل از جمله شیوع بالای بیماری‌های مزمن، توجه ویژه سیاستگذاران برای این گروه سنی و ادامه مراقبت‌های بهداشتی به طور جدی‌تر ضرورت دارد. همچنین، با توجه به این که وضعیت سبک و کیفیت زندگی سالمندان از سطح متوسط بالاتر است، از این‌رو، برنامه‌ریزی جامع و دقیق‌تر در خصوص آموزش شیوه‌های زندگی سالم و مراقبت‌های بهداشتی درمانی می‌تواند در ارتقای سبک و کیفیت زندگی سالمندان و سلامت آن‌ها مؤثر باشد.

Title: حذف فتوکاتالیستی سافرانیناز آب به وسیله بستر ثابت نانو‌‌‌‌ ذرات دی‌اکسید تیتانیم نیکل دوپ پوشش داده شده بر روی زئولیت طبیعی کلینوپتیلولیت

http://hsr.mui.ac.ir/index.php/jhsr/article/view/2246

مقدمه: دوپ کردن برخی از فلزات با فتوکاتالیست‌ها و افزایش سطح آن‌ها باعث افزایش کارایی فتوکاتالیستی آنان می‌شود. در مطالعه حاضر، از دوپنت نیکل و زئولیت طبیعی ایرانی برای افزایش کارایی نانو دی‌اکسید تیتانیم به منظور حذف فتوکاتالیستی ترکیبات آزونیوم از آب استفاده شد. روش‌ها: تیتانیم نیکل دوپ پوشش داده شده بر روی بستر زئولیت کلینوپتیلولیت با 4 نسبت 17/0، 2، 9 2/TiO Ni و 2TiO خالص به وسیله روش سل- ژل ساخته شد و با روش‌های آنالیز دستگاهی پراش پرتو ایکس (XRD یا X-ray splashing)، میکروسکوپ الکترونی روبشی (SEM یا Scanning electronic microscope) و پلاسمای جفت شده القایی (ICP یا Induced coupled plasma) مورد بررسی قرار گرفت. سپس، میزان کارایی فعالیت فتوکاتالیستی نسبت‌های مختلف دوپنت نیکل تحت پرتو فرابنفش (UV یا Ultra-violet ray) در غلظت‌های 25 و 50 mg/l سافرانین (ترکیب فنازینی) محلول در آب بررسی شد. یافته‌ها: در مطالعه حاضر، فازهای آناتاز و روتایل دی‌اکسید تیتانیم ساخته شده با روش سل- ژل با درصد فراوانی 33/86 درصد (اندازه 44/34 نانومتر) و 67/13 درصد (اندازه 18 نانومتر) مشاهده شد. مطابق نتایج، نمونه‌های حاوی دوپنت نیکل کارایی فتوکاتالیستی بهتری را نسبت به نمونه‌های فاقد نیکل از خود نشان داد. علاوه بر این، اضافه کردن نیکل باعث کاهش درصد فراوانی فاز آناتاز نمونه‌ها و همچنین، افزایش سرعت واکنش فتوکاتالیستی گردید. نمونه N9TC با کارایی حذف فتوکاتالیستی 93 و 79 درصد برای غلظت‌های 25 و 50 mg/l سافرانین بالاترین کارایی حذف فتوکاتالیستی را در بین سایر نمونه‌های فتوکاتالیستی داشت. نتیجه‌گیری: استفاده از زئولیت کلینوپتیلولیت ایرانی به عنوان بستر راهی مقرون به صرفه برای افزایش فعالیت فتوکاتالیستی می‌باشد. همچنین، اضافه کردن نیکل به دی‌اکسید تیتانیم باعث افزایش کارایی حذف فتوکاتالیستی سافرانین به عنوان یک ترکیب فنازینی می‌شود. افزایش نسبت Ni/TiO2 تا 9 درصد باعث افزایش کارایی حذف فتوکاتالیستی نمونه‌ها نسبت به نمونه‌های حاوی دی‌اکسید تیتانیم خالص شده است.

Title: سنتز الکتروشیمیایی فیلم متخلخل نانوذرات نیکل اکسید در محیط اسیدی: کاربرد در ساخت حسگر پارانیتروفنل

http://hsr.mui.ac.ir/index.php/jhsr/article/view/2366

مقدمه: پارانیتروفنل یکی از مهم‌ترین ترکیبات نیتروبنزنی است که جهت سنتز بسیاری از ترکیبات شیمیایی صنعتی به طور گسترده‌ای به کار می‌رود. این ماده برای مدت زمان طولانی در خاک، آب‌های زیرزمینی و هوا باقی می‌ماند و به دلیل سمیت، باعث صدمه به انسان‌ها، حیوانات و گیاهان می‌گردد. به منظور ارزیابی سلامت منابع آب و غذا، طراحی و توسعه روش‌های کارا، ساده و قابل اعتماد جهت اندازه‌گیری مقادیر ناچیز این ترکیبات، اهمیت زیادی دارد. روش‌ها: در پژوهش حاضر به منظور اندازه‌گیری مقادیر ناچیز پارانیتروفنل، برای اولین بار با ترسیب الکتروشیمیایی فیلم نانوساختار نیکل اکسید به صورت در جا در سطح الکترود کربن شیشه‌ای، حسگر بسیار ساده و حساسی برای پارانیتروفنل ساخته شد. ولتامتری چرخه‌ای و اسپکتروسکوپی امپدانس الکتروشیمیایی (Electrochemical Impedance spectroscopy یا EIS) جهت ارزیابی و بررسی فیلم ترسیب شده در سطح الکترود استفاده گردید. یافته‌ها: بر اساس یافته‌‌ها، فیلم ترسیب شده در سطح الکترود، خاصیت الکتروکاتالیزوری بسیار خوبی جهت احیای الکتروشیمیایی پارانیتروفنل داشت. با اصلاح سطح الکترود کربن شیشه‌ای با این فیلم، حسگر الکتروشیمیایی حساسی جهت اندازه‌گیری پارانیتروفنل به روش ولتامتری چرخه‌ای تهیه شد. حد تشخیص، حساسیت و محدوده خطی پاسخ حسگر برای شناسایی پارانیتروفنل به ترتیب برابر با 40 نانومولار، 3245/0 میکروآمپر بر میکرومولار و 40 تا 750 میکرومولار محاسبه گردید. نتیجه‌گیری: نتایج مطالعه حاضر نشان داد که حسگر طراحی شده با حساسیت بسیار بالایی، برای اندازه‌گیری پارانیتروفنل قابل کاربرد می‌باشد.

Title: ارزیابی تأثیر برنامه آموزشی سواد سلامت به کارکنان بهداشتی بر ارتقای خودکارامدی بیماران دارای بیماری مزمن

http://hsr.mui.ac.ir/index.php/jhsr/article/view/2415

مقدمه: سواد سلامت، ميزان ظرفيت هر فرد براي كسب، تفسير و درك اطلاعات اوليه از خدمات سلامتي برای اخذ تصميم‌گيري مناسب مي‌باشد. این مطالعه، با هدف تعیین تأثیر برنامه آموزش سواد سلامت ارتباطی به کارکنان مراکز بهداشتی- درمانی بر ارتقای خودکارامدی بیماران دارای بیماری مزمن در شهر مشهد اجرا شد. روش‌ها: پژوهش حاضر یک مطالعه تجربی از نوع مداخله قبل و بعد شاهددار تصادفي شده بود که بر روی 240 نفر از بیماران دارای بیماری مزمن به روش نمونه‌گيري تصادفی چند مرحله‌ای انجام گردید. مداخله آموزشی مبتنی بر سواد سلامت بر اساس چهار مهارت ارتباط گفتاری، نوشتاری، توانمندسازی و بهبود سیستم‌های حمایتی، طراحی و طی سه جلسه بحث گروهی متمرکز و یک کارگاه آموزشی برای 12 نفر از کارکنان بهداشتی (پزشک، پرستار،کارشناس تغذیه و بیماری‌ها) درگروه آزمون انجام شد. ابزار جمع‌آوري داده‌ها شامل پرسش‌نامه ارزیابی سواد سلامت، مراقبت اولیه در بزرگسالان و خودکارامدی در بیماری مزمن بود. داده‌ها با استفاده از نرم‌افزار SPSS و آزمون‌های t، ANOVA و 2χ با در نظر گرفتن سطح معنی‌داری کمتر از 5 درصد تجزیه و تحلیل گردید. یافته‌ها: بیماران در دو گروه از نظر متغیرهای فردی با یکدیگر تفاوت معنی‌داری نداشتند (063/ = P). بعد از مداخله، تفاوت آماری معنی‌دار در میانگین سواد سلامت کارکنان (007/0 = P)، سواد سلامت بیماران (001/0 > P) و خودکارامدی بیماران (003/0 = P) در گروه مداخله مشاهده گردید. نتیجه‌گیری: مداخلات آموزشی جهت ارتقای سواد سلامت ارتباطی کارکنان بهداشتی در گروه مداخله در ارتقای خودکارامدی بیماران در مقايسه با آموزش‌هاي رايج در مراکز بهداشتی- درمانی، مؤثرتر می‌باشد.

Title: بررسی کارایی فرایند سونوفتوکاتالیستی نانوذرات اکسید روی در حذف فرمالدهید از محلول‌های مایی

http://hsr.mui.ac.ir/index.php/jhsr/article/view/2433

مقدمه: مطالعه حاضر با هدف بررسی میزان تجزیه‌پذیری فرمالدهید توسط فرایند سونوفتوکاتالیستی اکسید روی، با استفاده از یک رآکتور اکسیداسیون به حجم تقریبی 2 لیتر انجام شد. روش‌ها: این مطالعه از نوع بنیادی کاربردی بود و به صورت مقطعی انجام گردید. در طی چند مرحله آزمایش (30 مرحله)، تأثیر متغیرهای مهم بهره‌برداری از جمله pH محلول (3 تا 8)، غلظت اولیه فرمالدهید (100-20 میلی‌گرم در لیتر)، دوز نانوذرات اکسید روی (60-10 میلی‌گرم در لیتر)، میزان قدرت امواج اولتراسونیک (150-40 وات) و زمان واکنش (60-10 دقیقه) بر روی میزان تجزیه فرمالدهید مورد بررسی قرارگرفت. یافته‌ها: حداکثر میزان حذف فرمالدهید در شرایط مطلوب شامل دوز نانوذرات اکسید روی 60 میلی‌گرم در لیتر، pH محلول برابر با 8، قدرت امواج اولتراسونیک 40 وات، غلظت اولیه فرمالدهید 50 میلی‌گرم در لیتر و مدت زمان واکنش 30 دقیقه بود. در این شرایط، کارایی حذف فرمالدهید حدود 78 درصد به دست آمد.نتیجه‌گیری: نتایج حاصل از مطالعه حاضر نشان داد که استفاده از روش سونوفتوکاتالیستی جهت تصفیه محلول‌های مایی حاوی غلظت کم فرمالدهید، مؤثر می‌باشد.

Title: بررسی وجود پروتئین گلوتن با استفاده از تکنیک الکتروفورز SDS-PAGE در نان‌های فاقد گلوتن تهیه شده جهت بیماران سلیاک در شهر اصفهان

http://hsr.mui.ac.ir/index.php/jhsr/article/view/2654

مقدمه: بیماری سلیاک یا انتروپاتی حساس به گلوتن، بیماری مرتبط با سیستم ایمنی است كه به صورت حساسیت دایم به گلیادین گندم یا سایر پرولامین‌های موجود در جو در افرادی كه از نظر ژنتیكی مستعد هستند، تعریف می‌شود. گلوتن پروتئینی است که در گندم، جو و چاودار یافت می‌شود. این ماده باعث کش آمدن خمیر و ترد شدن بافت فراورده نهایی می‌گردد. گلوتن ترکیبی از دو زیر واحد گلیادین و گلوتنین می‌باشد که گلیادین محلول در الکل است و گلوتنین فقط در باز و اسیدهای رقیق محلول می‌باشد و بر اساس این ویژگی‌ها می‌توان گلیادین را جداسازی کرد. با تعیین گلوتن موجود در آرد نان فاقد گلوتن مخصوص به بیماران سلیاک با روشی مناسب و دقیق، می‌توان صحت ادعای تولید کنندگان این محصولات را تعیین نمود. روش‌ها: نمونه مورد بررسی آرد نانوایی بود که از تعداد 24 نمونه آردی که برچسب فاقد گلوتن داشت، تهیه و از الکل ایزوپروپانل به عنوان حلال الکلی استفاده شد. در مرحله بعد، طبق روش الکتروفورز SDS- PAGE (Sodium dodecyl sulfate- Polyacrylamide gel) ترکیب استخراج شده پس از آماده‌سازی با بافر مخصوص و همچنین، نمونه گلوتن خالص به عنوان کنترل مثبت به ژل اکریل آمید تزریق گردید. پس از اتمام این مرحله، ژل توسط محلول رنگی کوماسی بلو رنگ‌آمیزی و سپس، با محلول رنگ‌بر شفاف‌سازی شد. یافته‌ها: ژل حاصل از این آزمون در مقایسه با نمونه شاهد مثبت و همچنین، مارکر پروتئینی استاندارد، باند مشخصی در محدوده مربوط به گلیادین بر روی ژل قابل رویت نبود که دلالت بر فقدان گلوتن در نمونه آرد داشت. نتیجه‌گیری: در تکنیک الکتروفورز SDS-PAGE به دلیل حساسیت و دقت بالا، مقادیر بسیار کم گلوتن قابل سنجش می‌باشد. بنابراین، تشخیص این مقادیر در آرد و خودداری از مصرف آن در بیماران مبتلا به سلیاک از بروز تظاهرات بالینی جلوگیری خواهد کرد. از مزایای دیگر این تکنیک سرعت بالا و کم هزینه بودن آن می‌باشد.

Title: تبیین عوامل رفتاری تسهیل کننده درمان بیماران مبتلا به سل: یک تحلیل کیفی

http://hsr.mui.ac.ir/index.php/jhsr/article/view/2036

مقدمه: مهم‌ترین منبع عفونت سل در جامعه، بیماران مبتلا به سل ریوی اسمیر مثبت هستند که اگر با موفقیت درمان نشوند و درمان خود را قطع نمایند، باعث شیوع سل مقاوم به درمان در جامعه می‌شوند. مداخلات درمانی بدون توجه به علل رفتارهای مرتبط با درمان، چندان موفق نخواهد بود. پژوهش حاضر به منظور تبیین عوامل رفتاری تسهیل کننده درمان بیماران مبتلا به سل انجام شد. روش‌ها: این مطالعه از نوع کیفی بود که در آن از نمونه‌گیری مبتنی بر هدف با حداکثر تنوع (Maximum variation) استفاده گردید. داده‌ها از طریق مصاحبه عمیق سازمان نیافته با 37 نفر (21 مرد و 16 زن)، مشاهده پرونده بیماران، اطلاعات ثبت شده در برنامه Tuberculosis register و دفتر ثبت‌نام بیماران مبتلا به سل در مراکز بهداشت شهرستان‌ها، جمع‌آوری شد. مصاحبه‌ها در یک بازه زمانی 13 ماهه تا رسیدن به اشباع داده‌ها ادامه یافت. تمام مصاحبه‌ها پس از ضبط به صورت کامل تایپ شد و داده‌ها با مقایسه مداوم و هم‌زمان با جمع‌آوری آن‌ها مورد تجزیه و تحلیل قرار گرفت. یافته‌ها: عوامل رفتاری تسهیل کننده درمان در 6 طبقه شامل «ترک استعمال مواد مخدر و دخانیات در طول درمان، متادون درمانی در طول درمان، مسافرت نرفتن در طول درمان، خسته نشدن و مصمم بودن در مصرف صحیح داروها تا اتمام دوره درمان، امید به بهبودی و سلامت عاملی برای تداوم درمان و عادی شدن مصرف داروها برای بیمار» دسته‌بندی گردید. نتیجه‌گیری: بر اساس نتایج مطالعه حاضر که حاصل آن شناسایی عوامل رفتاری مؤثر بر درمان بیماران مبتلا به سل بود، باید مداخلات آموزشی مناسبی جهت تقویت عوامل تسهیل کننده، به منظور آموزش مؤثر بر درمان بیماران طراحی و اجرا گردد.

Title: بررسی علل و روند 5 ساله مرگ و میر استان ایلام از سال 1386 تا 1390

http://hsr.mui.ac.ir/index.php/jhsr/article/view/2846

مقدمه: يكي از شاخص‌های مهم برای انجام برنامه‌ریزی‌های مربوط به سلامت جوامع، میزان و علل مرگ و میر در آن جوامع است. هدف از انجام مطالعه حاضر، بررسی علل و روند 5 ساله مرگ و میر استان ایلام در محدوده سال 86 تا 90 بود. روش‌ها: پژوهش حاضر، یک مطالعه مقطعی- توصيفی- تحليلی بود که بر روی داده‌های ثبت شده مرگ استان ایلام به تفکیک در مناطق شهری و روستایی از سال 86 تا90 انجام شد. داده‌های مورد نیاز مطالعه توسط یک فرم محقق ساخته از اطلاعات جمع‌آوری شده توسط واحدهای گسترش و آمار مرکز بهداشت استان (سیستم ثبت مرگ) و نیز از آمارهای مرگ اعلام شده توسط سازمان ثبت احوال استان جمع‌آوری شده و مهم‌ترین علل مرگ به تفکیک استخراج گردید. یافته‌ها: میزان مرگ در مناطق شهری و روستایی استان به ترتیب در سال 86، 3/3 و 2/5 در هزار و در سال 90 در حدود 6/4 و 6/3 در هزار گزارش شد. همچنین، میزان مرگ و میر در زنان طی این سال‌ها از 1/3 به 6/3 و در مردان از 8/4 به 9/4 رسید. بیشترین تعداد مرگ و میر در مناطق شهری و روستایی در گروه سنی 70 سال به بالا و بیشترین علت مرگ در استان، بیماری‌های قلبی و عروقی بود. نتیجه‌گیری: نتایج حاصل از مطالعه نشان داد که به ترتیب داخل شهر و روستا در سال 86، با 9/43 درصد و 36 درصد و در سال 90، با 9/52 درصد و 1/39 درصد ناشی از بیماری‌های قلبی و عروقی بود. در طی سال‌های مورد بررسی 86 تا 90، سیر مرگ به علت بیماری‌های قلبی- عروقی افزایش، سرطان‌ها کاهش، سوانح و حوادث دارای نوسان بوده و در مجموع تا حدودی کاهش، خودکشی تا حدودی کاهش، کهولت کاهش و سایر موارد نیز به طور تقریبی ثابت بود. از آن‌جا که ابتلا به این دسته از بیماری‌ها تا حدود زیادی وابسته به سبک زندگی است، به نظر می‌رسد که انجام برنامه‌ریزی لازم از طریق وضع قوانین و مقررات و نیز آموزش در خصوص عوامل خطرساز سلامت نظیر استعمال دخانیات، بی‌تحرکی، رژیم غذایی نامناسب، اضطراب و... در راستای بهبود سبک زندگی افراد، زمینه کاهش این دسته از بیماری‌ها را فراهم می‌سازد.

Title: ارتباط سواد سلامت با وضعیت سلامت کارکنان اداری دانشگاه علوم پزشکی گیلان

http://hsr.mui.ac.ir/index.php/jhsr/article/view/2580

مقدمه: امروزه سواد سلامت چالشی برای تأمین کنندگان مراقبت بهداشتی- درمانی و سیستم‌های سلامت می‌باشد و باید در تمام بخش‌های سلامت مورد توجه قرار گیرد. مطالعه حاضر ارتباط سواد سلامت را با وضعیت سلامت کارکنان اداری دانشکده‌های دانشگاه علوم پزشکی گیلان در سال 1394 بررسی کرد. روش‌ها: این مطالعه از نوع مقطعی- تحلیلی بود. کارکنان اداری دانشکده‌های دانشگاه علوم پزشکی گیلان به روش سرشماری وارد مطالعه شدند. جمع‌آوری داده‌ها از طریق پرسش‌نامه‌ای مشتمل بر سه بخش اطلاعات فردی، وضعیت سلامت و سنجش عملکردی سواد بزرگسالان (Health literacy for Iranian adults یا HELIA) صورت گرفت. داده‌ها با استفاده از آمار توصیفی و استنباطی در نرم‌افزار SPSS تجزیه و تحلیل گردید. یافته‌ها: سواد سلامت کلیه کارکنان (186 نفر) اداری خوب و متوسط به دست آمد. 81 نفر از کارکنان (8/44 درصد)، وضعیت سلامت خود را متوسط گزارش کردند. ارتباط آماری معنی‌داری بین وضعیت سلامت با تأهل (03/0 = P) و وضعیت اقتصادی (001/0 > P) و نیز بین سواد سلامت با تحصیلات (034/0 = P) و وضعیت بیمه (044/0 = P) مشاهده شد. بین سواد سلامت و وضعیت سلامت ارتباط معنی‌داری مشاهده نگردید. نتیجه‌گیری: سواد سلامت کلیه کارکنان اداری با توجه به نتایج، خوب و متوسط به دست آمد و مهم‌ترین عوامل مرتبط در امر سواد سلامت، داشتن تحصیلات و بیمه بود. از این‌رو، توجه به این متغیرها در ارتقای سطح سواد سلامت کارکنان اداری می‌تواند نقش بسزایی داشته باشد. پیشنهاد می‌شود که سیستم بهداشت و درمان با برنامه‌ریزی‌های آموزشی به حفظ سواد سلامت و نیز ارتقای آن در کارکنان اداری توجه ویژه‌ای نماید تا در نهایت موجب ارتقای سلامت عمومی و بهبود کیفیت خدمات آن‌ها گردد.
